# Bridging pathologies: Mechanistic insights into the diabetes–Alzheimer's nexus

**DOI:** 10.17179/excli2025-9165

**Published:** 2026-01-23

**Authors:** Aniket Kakkar, Harpreet Singh, Yash Jasoria, Arvind Kumar, Shivani Chopra, Hitesh Chopra, Arun Kumar Mishra

**Affiliations:** 1 SOS School of Pharmacy (Faculty of Pharmacy), IFTM University, Moradabad, Uttar Pradesh, 244102, India; 2 School of Pharmaceutical Sciences (Faculty of Pharmacy), IFTM University, Moradabad, Uttar Pradesh, 244102, India; 3 School of Medical & Allied Sciences, K. R. Mangalam University, Gurugram, Haryana, India; 4 Department of Biosciences, Saveetha School of Engineering, Saveetha Institute of Medical and Technical Sciences, Chennai, 602105, Tamil Nadu, India; 5 Centre for Research Impact & Outcome, Chitkara College of Pharmacy, Chitkara University, Rajpura, 140401, Punjab, India

**Keywords:** Type 2 diabetes mellitus, Alzheimer's disease, insulin resistance, oxidative stress, amyloid-beta, neurodegeneration

## Abstract

Type 2 diabetes mellitus (T2DM) is increasingly recognized as a major risk factor for Alzheimer's disease (AD), with mounting evidence highlighting shared pathophysiological mechanisms. This review explores the intricate biological and molecular links between these two chronic disorders. Key overlapping pathways include impaired insulin signaling, chronic inflammation, oxidative stress, mitochondrial dysfunction, amyloid-beta (Aβ) accumulation, tau hyperphosphorylation, and the formation of advanced glycation end-products (AGEs). Disruption of insulin signaling in the brain contributes to synaptic loss and neurodegeneration, while systemic metabolic disturbances aggravate blood-brain barrier dysfunction and neurovascular damage. Emerging studies also underscore the role of antidiabetic treatments, especially newer agents targeting the gut-brain axis, in modulating AD progression. The review further examines preclinical models, clinical observations, and the development of biomarkers to improve early detection and intervention. Despite growing insights, challenges remain in translating mechanistic knowledge into effective therapies. A multidisciplinary approach integrating metabolic control and neuroprotective strategies is essential for addressing the comorbid burden of T2DM and AD.

See also the graphical abstract(Fig. 1).

## Introduction

**Figure 1:**
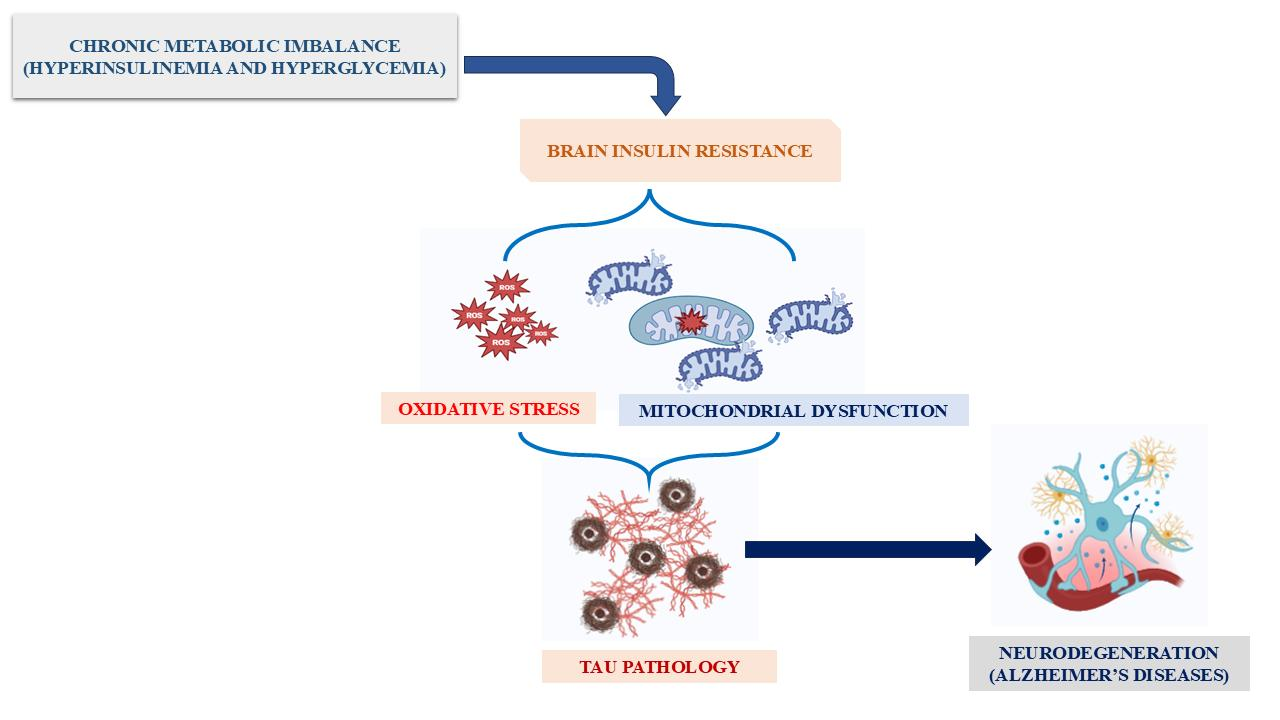
Graphical abstract

The global burden of Type 2 diabetes mellitus (T2DM) and Alzheimer's disease (AD) is growing at an unprecedented rate (Hamzé et al., 2022[98]). People with T2DM have an increased risk of developing AD compared to those with normal glucose tolerance, with large population-based cohort studies and meta-analyses indicating an approximately 1.4-2.0-fold elevated risk (Xue et al., 2019[273]). Notably, accumulating evidence suggests that T2DM and AD share overlapping clinicopathological features, including progressive neuronal injury and age-related neurodegenerative processes (Akter et al., 2011[8]; Stanciu et al., 2020[239]). In the central nervous system, insulin signaling plays a crucial role in cognition and the integrity of neural circuits (van der Heide et al., 2006[259]). The co-aggregation of beta-amyloid (Aβ) plaques is found to be promoted by T2DM and glucose hypometabolism-induced hypoxia (Chatterjee and Mudher, 2018[45]). These findings suggest that the relationship between T2DM and AD may be far more complex (Chauhan et al., 2024[47]). Several potential pathogenic pathways, such as reduced cerebral insulin sensitivity in T2DM, maternal diabetic milieu exposure, and microvascular rarefaction in T2DM, contribute to AD pathogenesis (Yang and Song, 2013[277]; Vieira et al., 2018[261]). Note that shared pathogenic mechanisms may represent suitable therapeutic targets for preventive AD treatments (Dai and Kamal, 2014[63]). The present review explores the growing evidence of the different molecular and cellular mechanisms underlying the association of T2DM with AD and examines the significance of such insights (Takeda et al., 2011[247]). In the subsequent section of the article, the mechanistic underpinnings of the late-life development of AD and the cognitive impairments observed in T2DM are laid out and triangulated (Luchsinger, 2012[147]). Moreover, the possibility that T2DM could induce an earlier onset of AD is investigated by addressing the time courses of cognitive decline and the rate of AD conversion in samples from Aβ-free individuals (Arvanitakis et al., 2004[16]; Sanz et al., 2009[223]). The concluding section of the article outlines potential directions for future research (Mittal and Katare, 2016[163]). It first argues the need to replicate the findings of the hypothesized mechanism linking AD to T2DM and AD to Aβ (Chatterjee and Mudher, 2018[45]). Subsequently, it provides alternative interpretations for the phenomenon of earlier AD onset in T2DM and considers potential explanations for the protective effects of T2DM against cognitive impairment and AD (Domínguez et al., 2014[71]; Sridhar, 2015[236]). The overarching molecular and cellular intersections between T2DM and AD, which are elaborated throughout this review, are schematically represented in Figure 1(Fig. 1).

## Methodology

A comprehensive literature search was conducted to identify relevant studies on the mechanistic link between T2DM and AD. The search was performed across multiple electronic databases, including PubMed/MEDLINE, Scopus, Web of Science, and Google Scholar. The search strategy employed a combination of Medical Subject Headings (MeSH) and Boolean operators to ensure an exhaustive and targeted retrieval of articles. The primary MeSH terms and keywords used included "T2DM," "AD," "Insulin Resistance," "Aβ," "Tau Pathology," and "Neuroinflammation." Boolean operators such as AND, OR, and NOT were utilized to refine the results. For instance, the query “(Type 2 Diabetes Mellitus OR T2DM) AND (Alzheimer's Disease OR AD) AND (Mechanisms OR Pathology)” was used to capture the biological and molecular interconnections between the two conditions. The inclusion criteria focused on peer-reviewed original research articles, systematic reviews, and meta-analyses published in English that investigated shared pathophysiological mechanisms, such as insulin signaling disruption, oxidative stress, mitochondrial dysfunction, amyloid and tau pathology, and advanced glycation end-products (AGEs). Both in vitro and in vivo studies, as well as clinical trials and observational studies, were considered. Articles solely discussing one disease without reference to the other or lacking mechanistic insights were excluded. Editorials, opinion pieces, and non-English publications were also excluded. Two independent reviewers screened the titles and abstracts of retrieved articles and selected full texts based on relevance and quality. A standardized data extraction form was used to collect key information, including study type, experimental models, biological targets, pathways discussed, and major findings. The findings were synthesized thematically and summarized in tables and figures to illustrate overlapping mechanisms between T2DM and AD. Additionally, molecular structures and biological terms were verified using NCBI MeSH and PubChem databases to ensure accuracy and consistency.

## Epidemiology of T2DM and AD

The prevalence of T2DM and AD has increased significantly over the past century, with a rapid increase in the number of individuals aged 65 years and older (Ribe and Lovestone, 2016[205]). To dissect an epidemiological relationship between two diseases like T2DM and AD, with a long prodromal period spanning several decades, the first series of studies averaged the A1c levels to study incident cognitive impairment (Mittal and Katare, 2016[163]; Zheng et al., 2018[286]). Subsequent longitudinal population-based studies have confirmed a bidirectional link between T2DM and cognitive decline, as well as an increased risk of incident AD, vascular and mixed dementia, and all-cause dementia (Cheng et al., 2012[49]; Wennberg et al., 2017[267]). Prospective cohort studies consistently report that individuals with T2DM have a significantly elevated risk of developing AD, with hazard ratios typically ranging from 1.4 to 1.6 and odds ratios approaching 2.0 in long-term follow-up studies (Zhang et al., 2017[282]; Jeong et al., 2024[120]). Importantly, these associations remain statistically significant after adjustment for major confounding variables, including age, sex, education, hypertension, dyslipidemia, obesity, cardiovascular disease, and lifestyle factors (Vagelatos and Eslick, 2013[258]). The magnitude of risk may be higher in women than in men and varies according to age, race, duration of T2DM, and history of severe hypoglycemia (Podcasy and Epperson, 2016[192]). Although partial attenuation of risk is observed after accounting for vascular comorbidities, the association between T2DM and AD generally persists, suggesting an independent contribution beyond cerebrovascular pathology (Lu et al., 2016[146]). Notably, some epidemiological studies report a later clinical onset of AD among Hispanic and non-Hispanic Black populations, which may reflect differences in genetic background, comorbidity burden, sociocultural factors, or diagnostic patterns rather than inherent biological resistance (Chin et al., 2011[52]; Santos et al., 2019[222]). Differences in T2DM duration may also affect other AD comorbid diseases, such as its association with age and glycemic control (Bruce et al., 2008[34]; Li et al., 2021[139]). However, Canadians may have a higher incidence of AD than Cubans, which may reflect geographical discrepancies in vascular disease and social status (Sanchez et al., 2024[220]). From a biological perspective, T2DM has a pivotal role in increasing the risk of developing AD even without comorbid disease, by altering Aβ protein clearance and accelerating the rate of hippocampal volume decline (Baglietto-Vargas et al., 2016[19]; Yadav et al., 2024[274]).

AD, the most prevalent cause of dementia, has shown a marked rise in incidence over the past century and is increasingly recognized as an independent risk factor contributing to cognitive decline (Reitz et al., 2011[204]; Tahami Monfared et al., 2022[245]). Some causes of this exponential epidemiological increase are likely due to the rapid increase in the lifespan of the species, but this does not explain the recent increase in AD in the absence of AD's risk factors, with a moderate genetic effect (Stozická et al., 2007[241]; Zhang et al., 2021[281]). Hypertension, dyslipidemia, heart disease, and T2DM are cardiovascular factors that are also known to increase the risk of developing cognitive decline and AD (Stampfer, 2006[238]; Grodstein, 2007[97]). In particular, T2DM has been shown to increase the risk of dementia: AD, vascular dementia, and mixed dementia, including T2DM (Exalto et al., 2012[79]). The wide variation in risk and incidence rates worldwide is believed to be due to sociodemographic and socioeconomic disparities, and the presence of a common stress factor, such as T2DM, is likely to aggravate the situation (Rizzi et al., 2014[207]; Baez et al., 2023[18]). In particular, the number of new cases of AD attributable to T2DM in late life is on the increase, given the dramatic rise in T2DM (Han and Li, 2010[101]). Finally, the life expectancy of subjects with T2DM has increased, which can also lead to an increase in the comorbidity of AD and T2DM (Moreira, 2012[167]).

## Common Pathological Mechanisms

T2DM and AD are two chronic, age-related diseases with increasing prevalence worldwide (Hamzé et al., 2022[98]). It is estimated that T2DM may more than double the risk of both cognitive impairment and AD (Domínguez et al., 2014[71]). Several common pathological mechanisms underlie the two diseases. Accumulating scientific findings suggest that these disorders possess similar etiological features regarding inflammation, metabolic dysfunction, and vascular impairment; thus, the link between them has become increasingly discernible (Jayaraman and Pike, 2014[119]; Li et al., 2015[142]). While there is no established cure or effective treatment for AD, it is believed that understanding the common mechanisms between T2DM and AD creates opportunities for prevention, early intervention, or disease modification for these severe disorders that have increased dramatically in the last decade (Mushtaq et al., 2014[169]; Vieira et al., 2018[261]).

**Figure 2:**
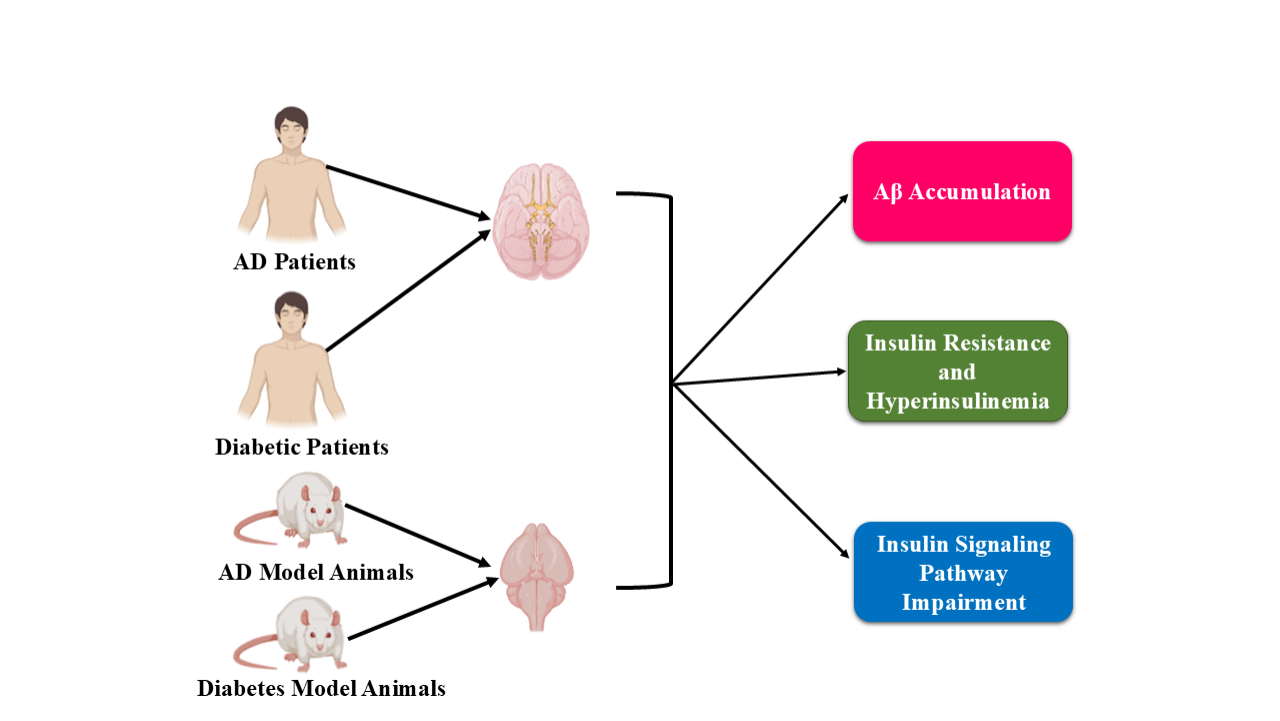
Common characteristics of AD and T2DM-related cognitive dysfunction (Tian et al., 2023)

Regarding the pathological mechanisms underlying AD and T2DM, several models have been proposed to establish the relationship between them, as shown in Figure 2(Fig. 2) (Reference in Figure 2: Tian et al., 2023[250]). The main findings indicate: (i) the synergistic accumulation of Aβ and tau in the animal models; (ii) the induction of Aβ in the pancreas of patients with T2DM and the induction of amyloidogenic proteins of the fibrillar type in the pancreas of AD patients; (iii) neurons present insulin resistance and glucose hypometabolism and are subsequently lost in both diseases; (iv) the insulin-degrading enzyme (IDE) shows similar activity in the brain of people with T2DM and AD (Wijesekara et al., 2017[268]; Busche and Hyman, 2020[36]; Patel et al., 2022[186]; Tian et al., 2023[250]). Overall, several chemical molecules seem to induce the activation of key molecular pathways involved in abnormal glucose metabolism and increase the pathology of AD (Chen and Zhong, 2013[48]). This may explain, at least in part, the epidemiological evidence that T2DM plays an important role in the development of AD (Ninomiya, 2019[177]). In summary, a better understanding of the mechanism responsible for the synergistic interaction between T2DM and AD will help us develop potential strategies for the integrated treatment and/or prevention of the two diseases (Takeda et al., 2011[247]; Hamzé et al., 2022[98]).

## Insulin Signaling Pathways in the Brain

**Figure 3:**
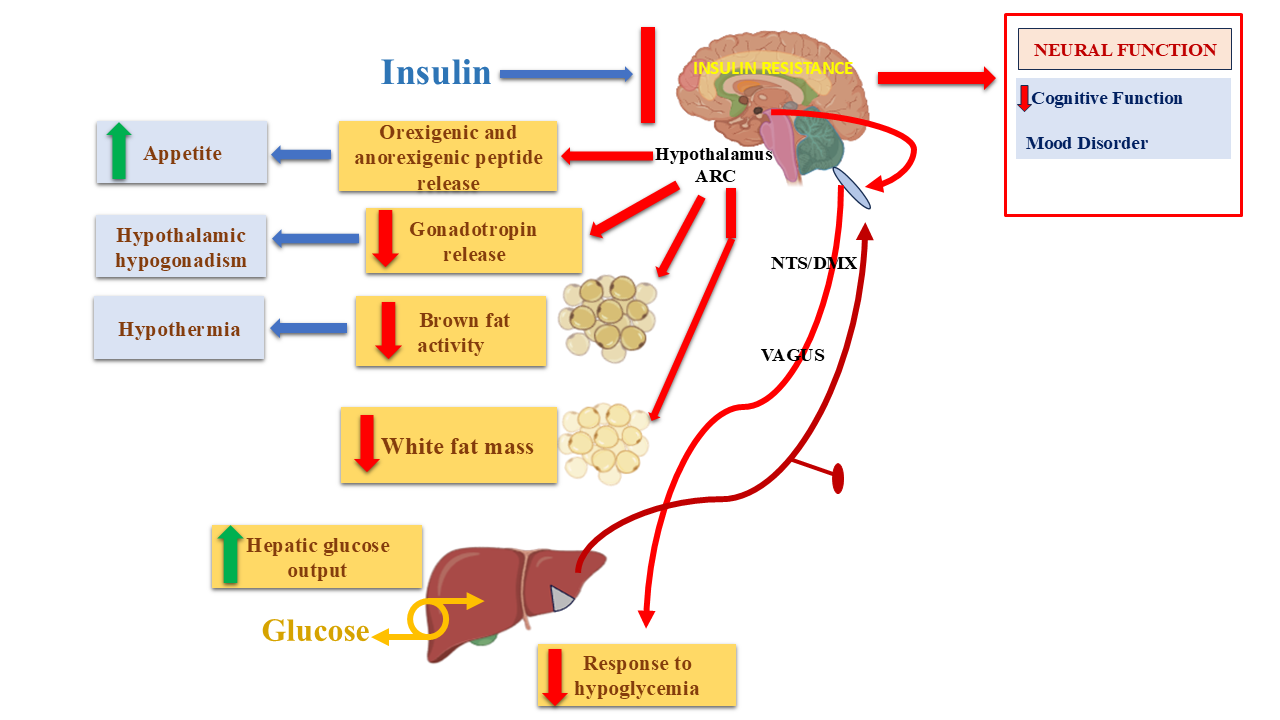
Insulin signaling in the brain has effects on both Peripheral and Central functions (Kleinridders et al. 2014)

The role of insulin signaling pathways has been studied primarily in the brain (Cole and Frautschy, 2007[58]). The primary action of insulin is on metabolism, but it also regulates the storage and metabolism of glucose, lipids, and other metabolites in the liver, peripheral tissues, and the brain (Figure 3(Fig. 3)) (Kleinridders et al., 2014[126]; Norton et al., 2022[178]). In the brain, insulin plays a critical role in regulating CO_2_ handling, cognition, perception, and reward in normal individuals (Dodd and Tiganis, 2017[70]). Insulin acts on the hypothalamus primarily, but also in the cerebral cortex (Zhao et al., 2004[285]). Insulin decreases lipolysis of adipocytes and thereby decreases lipids in the circulation. Hence, it is very clear that insulin plays diverse roles, depending on the region of action (Frühbeck et al., 2014[83]). The link between T2DM and clearance of intraneuronal Aβ by insulin has been understood (Tumminia et al., 2018[254]). Hence, AD and T2DM are known to have a bidirectional correlation (Xu and Shi, 2025[271]). Disruptions in insulin signaling pathways, a hallmark of T2DM, also occur in AD and form the mechanistic basis of this study (De Felice, 2013[66]). Hyperphosphorylation of the insulin receptor, but not insulin receptor substrate-1 on tyrosine residues, and Akt, and YWHAZ in mild cognitive impairment and AD brain contributes to synaptic dysfunction (Tanokashira et al., 2019[249]; Arvanitakis et al., 2020[15]; Zheng and Wang, 2021[287]). Aβ and p-Tau indirectly increase IRS1 and ERK phosphorylation by inhibiting PP2A (Torrent and Ferrer, 2012[252]). Hence, hyperphosphorylation of the insulin receptor is presumed to be a primary event, much before Aβ or tau accumulation begins (Rodriguez-Rodriguez et al., 2017[210]). Insulin and pioglitazone, when acting via the classical PI3K/Akt pathway, increase synaptic proteins and decrease Aβ proteins (Yang et al., 2017[276]; Gabbouj et al., 2019[85]). In addition, it has been observed that PI3K, Akt, PDK1, MAP kinases, and JNK are expressed before birth in the rat brain (Costa et al., 2016[60]). Tyrosine phosphorylation of insulin receptors begins in the hippocampus and midbrain in the fetus (Christie et al., 1999[55]). Neuraminidase 1, the rate-limiting enzyme in sialic acid degradation, is increased in insulin resistance (Dridi et al., 2013[73]). Insulin signaling has been shown to play a major role in neurogenesis (Gence et al., 2023[90]). The extent to which impairment of this pathway and its connection with other neural systems and glia is not known and has to be researched (Chung et al., 2015[56]). Hence, glucagon is not a viable option as a treatment strategy for AD or as a biomarker for AD (Wang and Ye, 2024[263]). The insulin signaling in the brain has also been discussed in the context of T2DM and AD (Hölscher, 2019[106]).

## Role of Inflammation in T2DM and AD

Low-grade chronic inflammation is a common feature of both T2DM and AD, highlighting the tight connection between them (Moyse et al., 2019[168]). In diabetic conditions, a grand puzzle of pro-inflammatory mediators is released by infiltrating immune cells at the level of the adipose tissue, creating a sort of pro-inflammatory environment permissive of insulin resistance and pancreatic damage (Odegaard and Chawla, 2012[180]; Burhans et al., 2018[35]). These mediators include cytokines, chemokines, adipokines, or metabolites able to propagate a pro-inflammatory message (Azizi et al., 2015[17]). The release of circulating pro-inflammatory mediators reaches the brain, even if accompanied in the aged and/or diabetic brain by a reduction of the anti-inflammatory potential of the blood-brain barrier (BBB) due to a down-regulation of some adenosine receptors, thereby favoring a further amplification of neuroinflammation (Chiu and Freund, 2014[53]; Takata et al., 2021[246]). It is remarkable that the interrelation of neuroinflammation with oxidative stress, another shared degenerative signal between T2DM and AD (Veselov et al., 2023[260]). An amplified pro-inflammatory environment can ultimately enhance the release of pro-inflammatory mediators arising from the damaged or stressed neurons, thus enhancing neuronal damage and ultimately the associated neurocognitive decline (Campbell, 2004[38]; García-Bueno et al., 2008[86]). Strikingly, patients with metabolic syndrome have also been found to show higher levels of pro-inflammatory mediators in the blood (Ma et al., 2012[153]).

The concept that the immune system communicates with the brain is further supported by the presence of receptor regulators of inflammation in the CNS found at the level of microglia, astrocytes, and neurons, and the increased release of glial pro-inflammatory mediators upon local damage or inflammation, or by the facilitated crossing of some circulating mediators at the level of the areas more highly permeable of the BBB (Becher et al., 2000[23]; Erickson et al., 2012[78]; Barcia et al., 2013[22]; Dong et al., 2014[72]). In turn, these pro-inflammatory mediators can interact not only with neurons to drive neuroinflammation and neurons' fate but also profoundly affect glia and neuron-glia crosstalk interactions, further propagating the inflammatory message (Skaper et al., 2018[232]; Bernaus et al., 2020[26]). Ongoing evidence suggests the presence of a tightly interconnected network of pro-inflammatory molecules released at the level of the adipose tissue with the brain, which can ultimately also impinge on the release of some factors (Aloe et al., 2015[11]; Parimisetty et al., 2016[184]). Neuroinflammation can exacerbate the progression of T2DM, while T2DM, in turn, amplifies neuroinflammatory responses, creating a vicious cycle that contributes to disease severity (Liyanagamage and Martinus, 2020[145]). Nonetheless, in the T2DM-associated enhanced circulating levels of the pro-inflammatory mediators, the onset occurring simultaneously with the manifestations of T2DM makes it quite difficult to distinguish between a possible causative role of T2DM or just a consequence of the neurological abnormalities (González-Reyes et al., 2016[95]; Sankar et al., 2020[221]). Nevertheless, the supported possibility that the T2DM-associated circulating molecules might elicit the type of neuroinflammation described, and if confirmed also in animal studies in the future, should generate new in vivo models of T2DM-associated cognitive decline in which the direct effect of T2DM-associated signals over neuroinflammation and subsequent neuronal damage can be explored and therapeutic approaches developed (Gaspar et al., 2016[87]; Rom et al., 2019[213]; Bellia et al., 2022[24]). Some implications arise from this association (Eikelenboom, 2016[75]). The most obvious is the possible exploitation of anti-inflammatory drugs developed or under study to prevent one disease in an attempt to avert the onset of the other (de Matos et al., 2018[67]). Consistent pediatric data now exist only in diabetic type-1 associated cognitive decline with increased inflammation (Gaudieri et al., 2008[88]). The pilot clinical studies that have already been performed seem to provide the scientific community with some encouraging data that deserve further investigation (Schwartz et al., 2014[226]). Such studies have investigated diagnostic and cure strategies, and more recently, diagnostic strategies (Brossaud et al., 2025[33]).

## Oxidative Stress and Mitochondrial Dysfunction

Oxidative stress is one of the hallmarks of rat models of T2DM as well as late-onset AD (Reddy et al., 2009[203]). The underlying mechanisms are pertinent. Increased oxidative stress results from increased production of reactive oxygen species (ROS) and subsequent failure of antioxidant defense mechanisms (Voronkova et al., 2018[262]). Oxidative stress also leads to decreased expression of nuclear-encoded genes for mitochondrial proteins, thus aggravating mitochondrial dysfunction (Mandelker, 2008[156]). Increased oxidative stress leads to increased oxidative damage to macromolecules by impairing the antioxidant mechanisms in various tissues and cellular fractions of the brain and insulin-responsive tissues such as skeletal muscle, liver, and pancreas (Henriksen et al., 2011[104]; Ahmad et al., 2017[5]). Accumulation of oxidative damage has been reported in neuronal tissues and is therefore termed a neurodegenerative process (Singh et al., 2019[229]). The oxidants are potent signal transducers that also mediate inflammation. The molecular routes that link oxidative stress with inflammation are pathways such as NF-κB (Lugrin et al., 2014[148]; Lingappan, 2018[143]).

Mitochondrial dysfunction is another important cellular process closely associated with neurodegenerative processes (Morais and De Strooper, 2010[166]). ROS induce damage to mitochondrial lipids, proteins, and nucleic acids, thus impairing mitochondrial functioning (Zia et al., 2022[289]). In turn, mitochondrial dysfunction leads to ROS production, creating a vicious cycle of progressively or rapidly aggravating neurodegenerative processes. Taken together, a clear synergistic interplay exists between oxidative stress and mitochondrial dysfunction (Bhat et al., 2015[27]; Islam, 2017[117]). Mitochondrial dysfunction also impairs glucose metabolism, confounding the treatment of T2DM-associated cognitive decline. Thus, protective neuronal strategies should focus on healthy mitochondria (Cheng et al., 2020[50]; Carvalho and Moreira, 2023[41]; Zhang et al., 2023[283]). Oxidative stress and inflammation are also major players in the development of insulin resistance and AD, as depicted in Table 1(Tab. 1). More likely, oxidative stress is central to a variable disease process, as further supported by the beneficial effects of antioxidants in the conditions of T2DM (Singh et al., 2024[230]).

## Aß and Tau Pathology

The accumulation of Aβ as plaques and hyperphosphorylated tau protein in NFTs are pathological hallmarks of AD (Huang and Jiang, 2009[112]). In healthy individuals, Aβ is produced when the amyloid precursor protein (APP), a transmembrane protein, is sequentially processed by β and γ secretase proteases, releasing Aβ peptides of 40 amino acids roughly every 50 seconds (Zhang et al., 2011[284]; Yuksel and Tacal, 2019[280]). Aβ peptides are produced throughout life, and while some remain soluble, a portion of them aggregate to form fibrils and plaques (Gouras et al., 2015[96]). Normally, the concentration of Aβ in the brain is maintained by clearance mechanisms, which include enzymatic degradation, cellular uptake via coated pits, and receptor-mediated transcytosis across the BBB (Yoon and Jo, 2012[278]). Aβ can recirculate into the vasculature and drain into the peripheral circulation via convective flow along perivascular pathways (Weller et al., 2008[266]).

Hyperphosphorylation of tau proteins, which constitute the microtubules that give neurons their shape, leads to reduced microtubule binding capacity, accumulation of paired helical filaments and NFTs, and it is estimated that 1.2-2.6 μmol of tau are released into the bloodstream each day (Iqbal et al., 2010[116]; Medeiros et al., 2011[159]; Alonso et al., 2018[12]). Importantly, Aβ and tau abnormal phosphorylation can occur in isolation, but together they colocalize and appear to promote each other in a feed-forward toxic cascade (Zhang et al., 2021[281]). Several lines of evidence point to dysregulation of Aβ processing and clearance in T2DM, fueling hope that early intervention in Aβ metabolism might hinder progression to Aβ pathology, particularly in cases of increased risk for AD (Maher and Schubert, 2009[154]; P et al., 2022[182]). In this regard, individuals with T1DM have higher concentrations of Aβ in cerebrospinal fluid, and the concentration of entorhinal cortex Aβ in older age is positively associated with insulin resistance and midlife non-insulin, but not insulin-dependent, glucose concentrations (Starks et al., 2015[240]; Hoscheidt et al., 2016[107]). Insulin acts primarily through IDE to degrade Aβ, and the risk of an individual with T2DM having MCI or dementia substantially increases with decreasing IDE activity (Qiu and Folstein, 2006[194]; Sun et al., 2016[243]). Several studies suggest that insulin resistance may also alter other Aβ clearance pathways, particularly those within the brain interstitial fluid (Craft, 2007[61]; Wei et al., 2021[265]). Given that insulin deficits, resistance, or desensitization impair non-amyloid metabolism in the brain, particularly synaptic and enteric health, it is also likely that insulinopathies contribute to the early formation and/or aggregation of Aβ, which in turn feed forward into other biological cascades, most notably tauopathy (Sabayan et al., 2008[218]; Matioli and Nitrini, 2015[157]; Rad et al., 2018[197]).

## Glycation and AGEs

Glycation is a non-enzymatic process of glucose-protein modification in which a reducing sugar forms reversible covalent crosslinks with the free amino groups present in protein or lipid molecules (Uceda et al., 2024[256]). Hyperglycemia accelerates the glycation process and results in adduct formation, with modification predominantly occurring in lysine, arginine, and hydroxylysine residues in proteins (Nawale et al., 2006[174]; Ahmed and Thornalley, 2007[7]). An Amadori rearrangement of Schiff base between the amino group of a protein or lipid molecule, followed by the formation of pyrroles, forms stable adducts referred to as AGEs (Yamagishi, 2011[275]; Twarda-Clapa et al., 2022[255]). AGEs formation can also result from the fragmentation of Amadori adducts, whether enzymatically at the glucosepane stage or non-enzymatically, and form crosslinks with other Amadori adducts or proteins to give rise to covalently protein-to-protein-crosslinked AGEs (Ulrich, 2001[257]; Goh and Cooper, 2008[93]). These modulated proteins can produce oligomeric structures called amyloidogenic protein oligomers (Takeuchi et al., 2004[248]). It is of considerable importance for protein structure and function to undergo glycation during the in vivo glycosylation process (Sirangelo and Iannuzzi, 2021[231]). Whereas protein glycation is considered unique, it can also be a common part of glycation-related chemical modifications, such as glycoxidation, lipid peroxidation, and reactions to form protein modifications of nevertheless forms of carbonyl (Lyons and Jenkins, 1997[152]). It ultimately ends in a dyschronogram of various proteins and underlying amino acid residues, which have profibrillogenic, procerogenic, and pro-inflammatory roles in various neurodegenerative conditions, including AD (Li et al., 2012[140]; Salahuddin et al., 2014[219]). The cellular downstream effects from the cell surface AGE-RAGE-oxothiolare system include the activation of the pro-inflammatory nuclear transcription factor-κB, the NLRP-3 inflammasome, and mitogen-activated protein kinases, the promotion of inflammation and an increase in oxidative stress activity, and eventually cell death and metabolic cell dysfunction (Fleming et al., 2011[81]; Tóbon-Velasco et al., 2014[251]; Chakraborty et al., 2021[44]). As a result, AGEs are the major contributory component to neuroinflammation and chronic oxidative stress that plagues AD patients, as depicted in Table 1(Tab. 1). The AGE-mediated crosslinking process also results in altered biophysical structures and functional properties of cellular proteins, and results in inorganoleptic protein appearance (Ulrich, 2001[257]). Given that AGE protein adducts play key roles in aging processes due to glycation, it is not difficult to make the case for research into these modifications in multiple pathophysiological domains' diagnoses (Fournet et al., 2018[82]; Rabbani and Thornalley, 2021[196]). Because food intake and metabolism are major contributors to these modifications, several groups have looked at AGE protein adducts about a variety of food-related health issues (Gill et al., 2019[92]; Cepas et al., 2020[43]). Further data will provide a more comprehensive understanding of the significance of these modifications in neuronal function and health (Kong et al., 2020[129]; Reddy et al., 2022[202]).

## Neurovascular Dysfunction and BBB Integrity

The BBB is composed of endothelial cells, astrocyte end-feet, pericytes, and the extracellular matrix of the basal lamina (Xu et al., 2019[272]). Maintaining the BBB structure and function is critical for establishing and maintaining the unique microenvironment within the central nervous system necessary for proper neuronal function (Abbott et al., 2010[2]). Numerous studies have established a link between BBB dysfunction and synapse and neuronal loss in the aging brain (Kurz et al., 2022[132]). The endothelium plays a critical role in the maintenance of vascular and BBB integrity (Engelhardt and Liebner, 2014[77]). In the context of T2DM, the integrity and function of this vascular component are compromised, leading to endothelial dysfunction (Roberts and Porter, 2013[208]). The consequences of T2DM on the vasculature have been shown to exacerbate age-related BBB and neuronal structure and function (Bogush et al., 2017[32]). It follows that restoring or protecting the integrity of the BBB has been posited as a potential therapeutic target in aging and neurodegenerative disease (Rust et al., 2025[217]).

If the BBB is dysfunctional or damaged, this can impair the neurovascular coupling, lead to reduced and slow cerebral blood flow, decreased clearance of waste products, and increased neuroinflammation (Zlokovic, 2011[291]; Obermeier et al., 2013[179]). Studies in humans and rodents reveal that BBB integrity is disrupted in the aging brain and worsened by T2DM, hypertension, and stroke (Goldwaser et al., 2016[94]). Indeed, brain trauma or pure cerebral ischemia leading to BBB disruption is associated with the development of neurological disorders such as progressive cognitive decline and increased dementia risk (Rosenberg, 2012[214]; Sweeney et al., 2018[244]). In support of the vascular hypotheses of cognitive decline, many studies have shown an association between decreased cerebral blood flow and clinical markers of cognitive decline, including impaired motor function and mood alterations, and dementia (Leeuwis et al., 2017[136]; Mokhber et al., 2021[164]). Based on these findings, pharmaceutical interventions have been implemented and shown to slow down disease progression in dementia patients (Hussain et al., 2021[114]). These studies suggest that the efficacy of anti-dementia drugs could rely on their ability to act as modulators of neurovascular function, thus confirming the vascular hypothesis (Bhat, 2015[28]).

## Impact of T2DM Treatments on AD Risk

**Figure 4:**
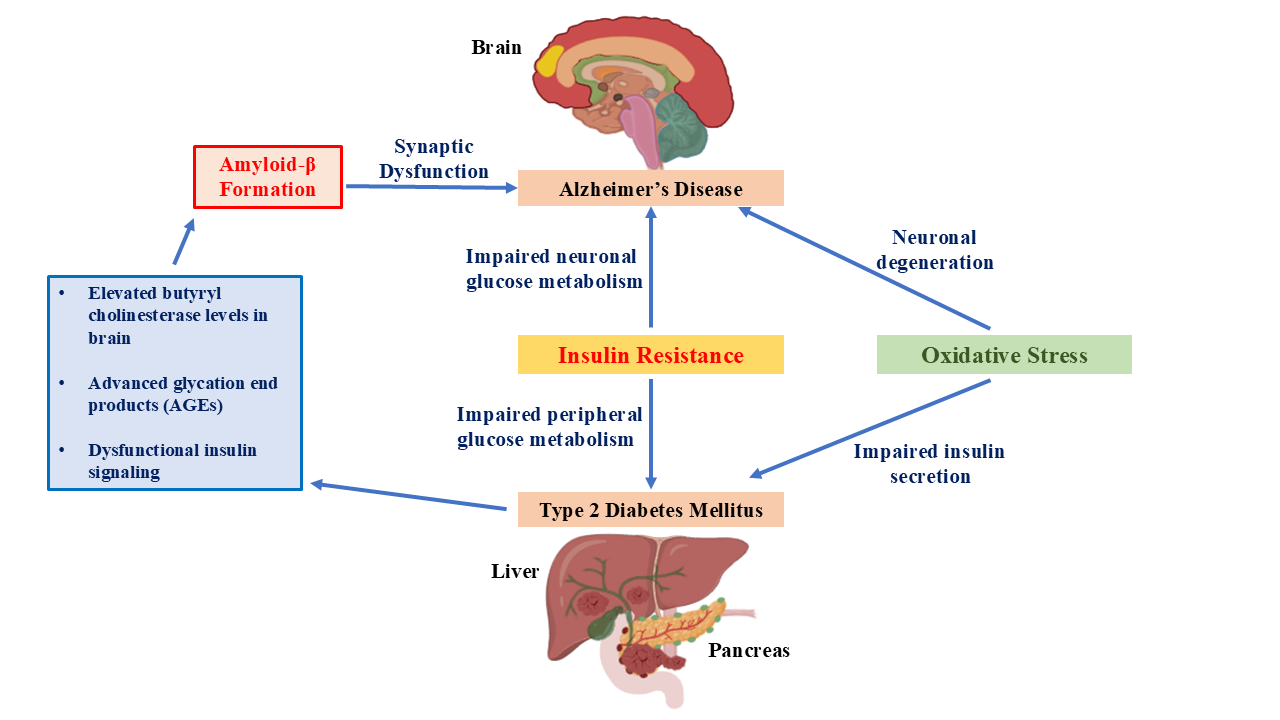
AD and T2DM may have similar pathogenic pathways

The far-reaching impact of T2DM has driven numerous studies to investigate the various scenarios, including molecular pathways and extensive molecular mechanisms that unite T2DM and AD (Figure 4(Fig. 4)). Classic old-generation treatments for T2DM, including sulfonylureas, metformin, and insulin therapy, have also been suggested at times to influence a slide towards AD through mechanisms such as dysregulated insulin signaling, toxicity of metformin, and accumulation of amyloidogenic peptides induced by prolonged insulin-based therapy (Boccardi et al., 2019[31]; Lynn et al., 2022[151]). The post-2007 dominance of the new generation of antidiabetic drugs, primarily acting on the incretin gut-brain axis, indicates a shift in the treatment of cognitive dysfunction (Tran et al., 2024[253]). Neprilysin (NEP), an amyloid-degrading metalloenzyme highly expressed in the kidney, has been shown to have an active glucose-lowering therapeutic role for inhibitors of sodium-dependent glucose transporter 2 (SGLT2) inhibitors, which also aid GLP, endocellular sodium, and water reabsorption (Peene and Benhalima, 2014[189]; AlAnazi et al., 2023[10]). Epidemiological studies and clinical trials have investigated the associations and roles of these new therapies with AD risk and progression (El-Amouri et al., 2008[76]; Nalivaeva et al., 2020[172]; Mancinetti et al., 2023[155]).

Results show that treatment with SGLT2 inhibitors could be efficacious in neuroprotection, and unexpectedly, the relation between Aβ and cognition is not linked to T2DM control, as evidenced by the lack of relationship between cognitive health and HbA1c (Raji, 2017[198]; Pawlos et al., 2021[188]). In line with this, scaling evidence also consolidates the absence of a relationship between HbA1c and the increase in neurodegeneration in hyperglycemia (Byun et al., 2017[37]; Fatih et al., 2022[80]). In conclusion, by addressing only T2DM control aspects, the potential training of the brain and neuroprotective effects of T2DM drugs need to be considered as part of the special link between T2DM and chronic neurological changes (Hu et al., 2024[108]). This could be of particular interest for people who depend more on glycemic control drugs to manage day-to-day glucose levels in proportion to non-pharmacologic methods (Monami et al., 2021[165]; Luo et al., 2023[149]). Relaxing the strict pathogenetic bias of the drugs' potential mechanisms behind the anti-diabetes medications that could affect dementia risk is essential because successful dementia risk reduction depends on increasing drug compliance as a cornerstone (Wium-Andersen et al., 2019[269]; Ogura and Yamaguchi, 2022[181]).

## Therapeutic Strategies Targeting the T2DM-AD Link

The convergence of T2DM and AD pathophysiology has prompted the exploration of therapeutic strategies that target shared molecular and cellular mechanisms, particularly insulin resistance, neuroinflammation, mitochondrial dysfunction, oxidative stress, Aβ accumulation, tau hyperphosphorylation, and AGEs (Rojas et al., 2021[212]; Lynn et al., 2022[151]; Han, 2024[100]). Traditional antidiabetic therapies such as insulin and metformin have demonstrated limited and conflicting effects on cognitive outcomes (Adem et al., 2024[3]). While insulin therapy may enhance brain insulin signaling and promote synaptic maintenance, chronic peripheral hyperinsulinemia can upregulate amyloidogenic pathways and competitively inhibit IDE, leading to increased Aβ accumulation (Zhao et al., 2004[285]; Qiu and Folstein, 2006[194]). Metformin has shown neuroprotective properties in some studies by activating AMP-activated protein kinase (AMPK) pathways, yet concerns remain regarding its potential to impair mitochondrial function and elevate the risk of lactic acidosis in vulnerable populations (Chiang et al., 2016[51]; Demaré et al., 2021[68]).

The emergence of next-generation antidiabetic agents, particularly SGLT2 inhibitors and GLP-1 receptor agonists, has opened new avenues in AD therapeutics (Złotek et al., 2023[292]). GLP-1 receptor agonists, such as liraglutide and exenatide, exert pleiotropic effects by enhancing insulin sensitivity, reducing neuroinflammation, modulating microglial activation, and promoting neurogenesis and synaptic plasticity (McClean et al., 2010[158]; Kopp et al., 2022[131]). Notably, these agents have demonstrated the ability to cross the BBB and directly act on central GLP-1 receptors (Katsurada and Yada, 2016[124]). SGLT2 inhibitors, originally developed to promote renal glucose excretion, are now recognized for their systemic anti-inflammatory properties, oxidative stress reduction, and potential to preserve cognitive function, independent of glycemic control (Pawlos et al., 2021[188]; Mei et al., 2024[160]). NEP, an endogenous Aβ-degrading enzyme, is being explored as a therapeutic target, with studies suggesting that NEP-enhancing strategies may facilitate Aβ clearance and ameliorate plaque burden (Fukami et al., 2002[84]; Saxena et al., 2024[224]).

In parallel, therapeutic efforts targeting tau pathology include kinase inhibitors (e.g., GSK-3β inhibitors), tau aggregation inhibitors, and immunotherapies designed to neutralize toxic tau species (Gerson and Kayed, 2016[91]). Since hyperphosphorylated tau is closely linked to neuronal dysfunction and is amplified by insulin resistance, tau-directed therapies are particularly relevant in T2DM-associated AD (Congdon and Sigurdsson, 2018[59]; Hobday and Parmar, 2021[105]). Furthermore, interventions aimed at mitigating AGEs formation or blocking AGE-RAGE signaling pathways are gaining attention, as AGEs contribute to sustained oxidative stress, inflammatory signaling, and protein crosslinking that exacerbate neurodegeneration (Srikanth et al., 2011[237]).

Addressing mitochondrial dysfunction and oxidative stress is also central to therapeutic development (Bhatti et al., 2017[29]). Agents such as coenzyme Q10, alpha-lipoic acid, and various polyphenols (e.g., resveratrol, curcumin) have shown promise in preserving mitochondrial function, reducing ROS production, and enhancing cellular resilience in preclinical models (Pagano et al., 2020[183]; Qin et al., 2024[193]). Anti-inflammatory therapies that inhibit proinflammatory cytokines (e.g., IL-1β, TNF-α), NLRP3 inflammasome activation, or toll-like receptor signaling may further alleviate neuroinflammation linked to both T2DM and AD (Mushtaq et al., 2015[170]; Söderbom and Zeng, 2020[234]).

Finally, non-pharmacological strategies remain indispensable. Caloric restriction, adherence to anti-inflammatory diets (such as the Mediterranean or MIND diets), regular physical activity, and cognitive engagement have been shown to improve insulin sensitivity, reduce systemic inflammation, enhance cerebral perfusion, and delay cognitive decline (Lautenschlager et al., 2014[135]; Daulatzai, 2017[65]). These lifestyle interventions are especially valuable due to their broad accessibility and capacity to modify multiple risk factors simultaneously (Klimova et al., 2017[127]). Future therapeutic paradigms should emphasize a multidomain approach, integrating precision pharmacotherapy with personalized lifestyle interventions, to effectively mitigate the shared trajectory of T2DM and AD (Alam et al., 2016[9]).

## Preclinical Models for Studying the Link

Over the last two decades, limited evidence has advanced from preclinical models for the mechanistic link between T2DM and AD (Mushtaq et al., 2014[169]; Lemche et al., 2024[138]). A wide range of animal models with T2DM-AD co-pathology are contributing to the field (Park, 2011[185]). Current preclinical models for studying the relationship between T2DM and AD include xenobiotic models, genetic models, Tg2576/SF1 mice, and Aβ infusion, as shown in Table 2(Tab. 2). Each model has advantages and shortcomings compared to the others, and not all of them are privileged to demonstrate learning and memory competence (Baglietto-Vargas et al., 2016[19]; Carranza-Naval et al., 2021[40]). Nevertheless, with these now readily accessible tools, studies on intracellular cross-talk between T2DM and AD may draw some conclusions (Rodriguez-Casado et al., 2025[209]). It is argued that the use of resistant mouse backgrounds, un-inbred, and female mice in these relevant models might better recapture the extensive spectrum of T2DM-related changes (Hardy et al., 2022[103]; Chauhan et al., 2024[47]).

Many studies using the models mentioned above have shown changes in learning and memory, brain volume, fibrillary Aβ, inflammation, tau, or synapse markers that are represented in the preclinical neuropathology of AD and T2DM, all of which characterize the co-pathology of either T2DM or HFDM with AD (Infante-Garcia et al., 2016[115]; Ramos-Rodriguez et al., 2017[199]; Wijesekara et al., 2017[268]; Chatterjee and Mudher, 2018[45]). In addition, it is very challenging to use the existing evidence to demonstrate the cause-and-effect relationship of T2DM or HFDM on AD with these models (Karki et al., 2017[121]). Furthermore, since some of them are not hereditarily AD-types or compromised AD-type animals that develop other side effects, their use in test therapy specifically directed towards AD is limited (Laurijssens et al., 2013[134]; Wang et al., 2024[264]). To conclude, these animal models may contribute to deciphering the complex mechanism responsible for the link between T2DM and AD, which is essential for the advancement of treatment in this field (Park, 2011[185]; Wijesekara et al., 2017[268]). It is important to expand the translational research from bench to bedside to improve the understanding of these relationships in clinic-based studies (Cummings et al., 2022[62]). Some emerging preclinical studies focus on mechanistic approaches and cutting-edge detection methods that may help to elucidate the complexity of this relationship (Gauthaman et al., 2014[89]).

## Clinical Studies and Observational Data

Numerous clinical studies and observational data are demonstrating an association between T2DM and AD, although these findings vary in methodological rigor (Schilling, 2016[225]). A recent review presents results from various cohort studies demonstrating that T2DM is associated with a high risk of developing AD (Huang et al., 2014[111]). Although these studies were conducted using heterogeneous participants and had a varying focus, the results seem concordant (Kopf and Frölich, 2009[130]; Zhang et al., 2017[282]). The methods used in these studies are typical of most observational studies and include detailed clinical assessments and neuropsychological evaluations (Paul et al., 2018[187]). In particular, participants were diagnosed with incident AD using clinical assessments and confirmatory postmortem neuropathological examination data, revealing the great pathogenic significance of risk factors associated with AD (A Armstrong, 2019[1]; Silva et al., 2019[227]).

Plausible demographic confounders of the observational T2DM-AD association include age, sex, and lifestyle risk factors, including inactivity, depression, and low educational status (Arvanitakis et al., 2004[16]; Nianogo et al., 2022[175]). The result of these variations is that the association between T2DM and AD can either be confounded by other risk factors (Vagelatos and Eslick, 2013[258]). Recent longitudinal studies also suggest that the T2DM-to-AD link may fluctuate over time (Lemche et al., 2024[138]). Observational studies have consistently reported an association between T2DM and an increased risk of developing AD; however, the relationship is not strictly linear, and factors such as disease duration, glycemic control, and coexisting vascular conditions may influence the strength of this association (Paul et al., 2018[187]; Celis-Morales et al., 2022[42]; Luo et al., 2023[150]; Chauhan et al., 2024[47]). Residual confounding and misclassification between AD and mixed or vascular dementia remain important methodological challenges; nevertheless, biomarker-supported studies increasingly indicate that T2DM contributes to AD-specific pathological processes (Li and Huang, 2016[141]; Chornenkyy et al., 2019[54]). Notably, a limited number of follow-up analyses have reported no significant association between T2DM and AD, highlighting heterogeneity across study designs and populations (Han et al., 2025[99]). Clinical trials are lacking (Baglietto-Vargas et al., 2016[19]). However, there are some modifiable risk factors that we addressed in this review (Myint, 2013[171]). A better understanding of the relationship between T2DM and AD is still possible (Nicolls, 2004[176]). The strongest evidence for causation comes from data that derives from a robust clinical trial (Kopf and Frölich, 2009[130]).

## Biomarkers for Early Detection and Monitoring

AD is the most common form of dementia and a fatal neurodegenerative disorder, showing progressive cognitive impairment (Knopman et al., 2021[128]). Major damage to the brain has already occurred by the time clinical diagnosis of AD is made (Quiroz et al., 2011[195]). In dementia, AD is the main pathology responsible for 60-80 % of cases (Rostagno, 2022[215]). In the USA, the estimated prevalence of all-cause dementia is around 13 % (Plassman et al., 2007[191]). Given that the chances of being diagnosed with T2DM are nearly 60 % higher in people with dementia, it is important to recognize these patients (Chatterjee et al., 2016[46]). However, this can be challenging due to the progressive and age-related nature of AD (Simó et al., 2017[228]). Patients, therefore, generally report T2DM as a secondary condition, indicating that T2DM lowers the age of dementia onset (Zilkens et al., 2013[290]). A combination of fasting glucose and glycated hemoglobin can be used to diagnose T2DM and impaired glucose tolerance (Hu et al., 2010[109]). Evidence suggests that T2DM and AD share common risk factors involving insulin resistance, inflammation, oxidative stress, the build-up of protein aggregates, and the dysregulation of other responsible peptides in brain metabolism (Dai and Kamal, 2014[63]; Michailidis et al., 2022[161]).

To this end, many other biological substances, including proteins and metabolites, have been proposed as potential biomarkers of disease progression (Navas-Carrillo et al., 2021[173]). Ideally, a biomarker would be informative of signaling changes in cognitive function in those with established T2DM and even in those with normal glucose levels (Ehtewish et al., 2022[74]). Some of the research studies have focused on identifying biomarkers that can detect changes in structure and function within the brain, before any overt cognitive symptoms appear (Raskin et al., 2015[201]). This would provide an opportunity for a T2DM treatment to slow down their progression to AD (Biessels et al., 2020[30]). Other analyses have emphasized multidimensional profiles using innovative imaging techniques and imaging-based and wearable cognitive assessments, as well as measures of plasma and cerebrospinal fluid (Lehallier et al., 2016[137]; Roe et al., 2023[211]; Wright et al., 2024[270]). All these complex measurements are collectively referred to as liquid biopsies (Soelter et al., 2022[235]). After researchers and statisticians have developed reliable models and validated these models in independent samples or other cohorts, the next critical step is to integrate this biomarker information into routine clinical practice or validate the findings in clinical trial settings (Hansson et al., 2023[102]; Hunter et al., 2025[113]). In an ideal world, both the therapy and the biomarker should be used in a holistic manner, to manage the onset of T2DM- and/or obesity-associated AD in a timely and efficient manner (Khan and Hegde, 2020[125]). Despite the development of these biomarker models, they have limitations (Ball et al., 2025[20]). To date, there remains an urgent need to standardize the method of detecting the presence of T2DM-related AD and to identify the most relevant and specific protein and metabolite biomarker signatures suitable for their operational analysis and diagnosis (Diniz Pereira et al., 2021[69]; Liu et al., 2024[144]).

## Challenges and Future Directions

Methodological and conceptual challenges currently limit definitive knowledge of the links between T2DM and dementia, and particularly the risk for and progression of AD and its relationship to the common comorbidities associated with T2DM, including cardiovascular disease and depression (Bello-Chavolla et al., 2019[25]; Cao et al., 2024[39]). In addition, no studies have yet been reported that treatment of cognitive dysfunction can halt the progression of dementia, and few studies have attempted to examine the relationship between the different types of AD and T2DM (Areosa Sastre et al., 2017[14]; Perng et al., 2018[190]; Dao et al., 2023[64]). Large, complex studies are required that can isolate causality from ups and downs in cognitive decline, including whether the development of the more detailed hyperlipidemias in T2DM is related to AD onset (Strachan et al., 2008[242]; Ansari and Sawane, 2024[13]). Recruitment and selection of AD and T2DM subjects is challenging; the nature of cognitive impairment at the point of recruitment can make dementia sufferers less self-aware (Karran et al., 2019[122]). Similarly, many individuals are diagnosed early and may live with T2DM for up to a decade before developing complications such as cognitive decline or AD (Clark et al., 2000[57]). Simple analyses can miss subtle relationships, and yet AD and T2DM are diagnosed based on clinical symptoms or low specificity imaging methods that can complicate results (Ahmed et al., 2014[6]; Mirza et al., 2014[162]). A comprehensive multidisciplinary approach is imperative, integrating the genetic underpinnings of T2DM and aging, the metabolic dysregulation associated with hyperglycemia and related pathophysiological features, and the neurobiological mechanisms of the central nervous system implicated in the onset and progression of cognitive impairments observed in comorbid T2DM and dementia (Aderinto et al., 2023[4]; Yu et al., 2025[279]; Zhou, 2025[288]). Large-scale, cutting-edge analyses by consortia of genetic associations, epidemiological data, and metabolic changes in brain signals are invasive, difficult, and expensive to perform, even if they are set up in the short term as innovative, nested study designs (Barbagallo, 2014[21]; Laske et al., 2015[133]; Hu et al., 2020[110]). Health policy changes as a result of global aging and the increasing impact of AD on the health economy make this a necessity, and the current interest in the area by potential partners gives some hope that progress can be made (Riggs, 2001[206]; Rapp, 2010[200]). However, this span of complexity, innovation, and long-term progression contrasts with the need to achieve immediate therapeutic outcomes and to develop strategies that can rapidly impact patient treatment and care or introduce novel methodologies (Katsenos et al., 2022[123]; Michailidis et al., 2022[161]). Partnerships between academic, industry, funding bodies, and societies need to be recognized actively, assessed, and fostered, especially those that encourage and support collaboration between basic researchers, clinicians, and those working on the public health or educational end of the dementia care remit (Ivinson et al., 2008[118]; Snyder et al., 2018[233]; Roth et al., 2025[216]).

## Conclusion

The intricate interplay between T2DM and AD is underpinned by a convergence of pathophysiological mechanisms, including insulin resistance, chronic inflammation, oxidative stress, mitochondrial dysfunction, Aβ accumulation, tau hyperphosphorylation, AGEs, and neurovascular impairment. These overlapping pathways not only contribute to the onset and progression of both conditions but also highlight common therapeutic targets. Traditional antidiabetic therapies and emerging agents such as SGLT2 inhibitors and GLP-1 receptor agonists have shown potential in modulating neurodegenerative changes, although further research is needed to determine long-term efficacy and safety. Despite growing insights from preclinical and clinical studies, major challenges remain in establishing causality, identifying robust biomarkers, and translating mechanistic findings into clinical interventions. A multidisciplinary, precision-based approach that integrates pharmacological strategies with lifestyle modifications offers the most promising path forward. Continued investment in longitudinal studies and innovative models will be critical to unravel the complex biology of the T2DM-AD link and to develop effective, targeted prevention and treatment strategies.

## Notes

Aniket Kakkar and Hitesh Chopra (Centre for Research Impact & Outcome, Chitkara College of Pharmacy, Chitkara University, Rajpura, 140401, Punjab, India; E-Mail: chopraontheride@gmail.com) contributed equally as corresponding author.

## Declaration

### Acknowledgements

The authors are thankful to their respective parent institutions.

### Conflict of interest

The authors declare no conflict of interest.

### Authors' contributions

Aniket Kakkar: Conceptualization, Methodology, Supervision, Writing - Original Draft.

Harpreet Singh: Investigation, Data Curation, Writing - Review & Editing.

Yash Jasoria: Formal Analysis, Visualization, Validation.

Arvind Kumar: Resources, Literature Review, Writing - Review & Editing.

Shivani Chopra: Project Administration, Writing - Review & Editing.

Hitesh Chopra: Supervision, Writing - Original Draft, Writing - Review & Editing, Correspondence.

Arun Kumar Mishra: Software, Data Curation, Literature Review.

### Artificial Intelligence (AI) - assisted technology

No artificial intelligence tools were utilized in the preparation of this manuscript. Grammarly was employed for language correction and refinement. The authors have thoroughly proofread the manuscript and take full responsibility for the accuracy, integrity, and originality of its content. No AI tool was used for generating, analyzing, or interpreting scientific data or conclusions.

## Figures and Tables

**Table 1:**
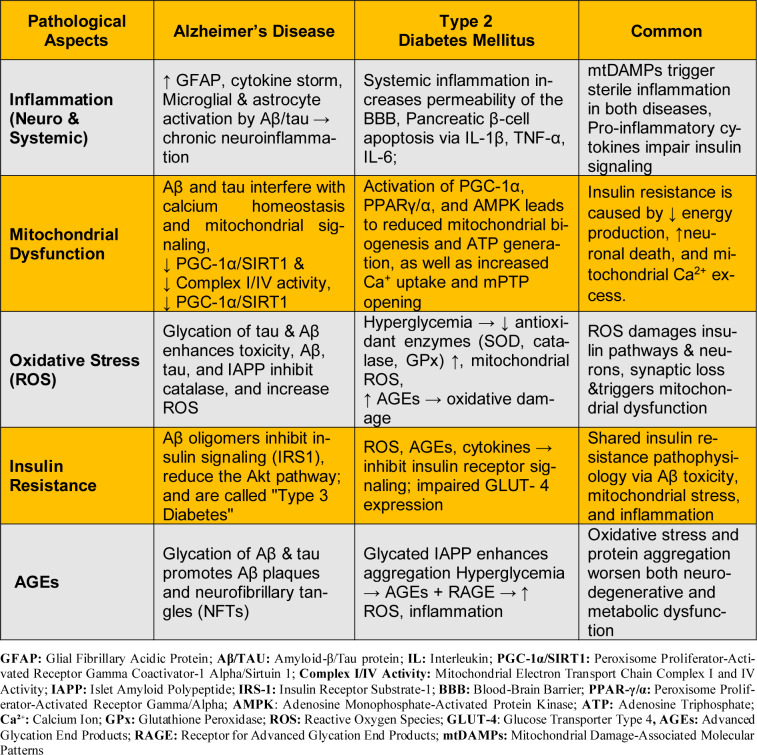
The relationship between oxidative stress, inflammation, and mitochondrial dysfunction in T2DM and AD

**Table 2:**
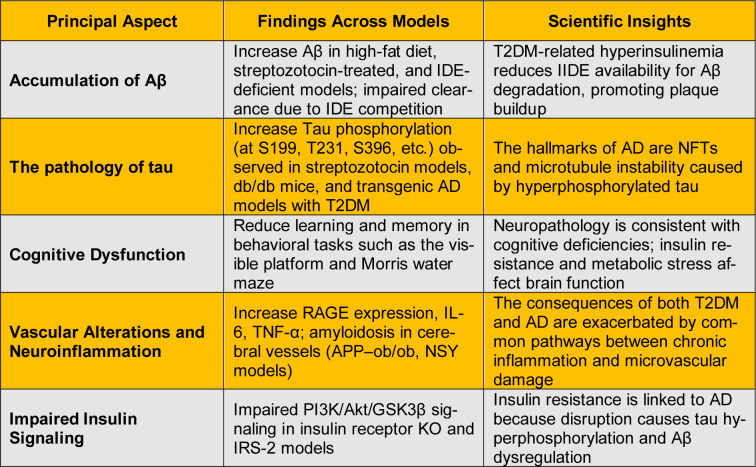
Common findings from animal models of T2DM and AD
